# Effects of N95 Masks Versus Surgical/Loop Masks on Arterial Blood Gases and Adverse Symptoms in Operating Room Staff During Prolonged Usage: A Pilot Study

**DOI:** 10.7759/cureus.69655

**Published:** 2024-09-18

**Authors:** Sandra Catanzaro, William Lavelle, W. Jacob Lavelle, Elizabeth A Demers Lavelle

**Affiliations:** 1 Anesthesiology, State University of New York Upstate Medical University, Syracuse, USA; 2 Orthopedic Surgery, State University of New York Upstate Medical University, Syracuse, USA; 3 Medicine, Colgate University, Hamilton, USA

**Keywords:** blood oxygen saturation (spo2), loop/surgical mask, n95 mask, partial pressure of carbon dioxide (pco2), partial pressure of oxygen (po2), transcutaneous partial pressure carbon dioxide (tcpco2)

## Abstract

Introduction: The COVID-19 pandemic continues to have a catastrophic impact on the global population. N95 masks are commonly used as filtering facepiece respirators for healthcare workers. At the end of long shifts, they have reported headaches, dizziness, fatigue, exhaustion, and physical and mental discomfort. There is a lack of data on the effects of N95 masks on blood gases in healthcare workers who wear masks for longer durations. We analyzed and compared the effects of continuously wearing N95 versus loop/surgical masks on various symptomology parameters and arterial blood gases for longer durations.

Methods: This was a non-blinded, pilot, observational study at a single academic institution. Utilizing a survey, we collected information on operating room staff volunteers (demographics, mask use, and symptoms) and measured transcutaneous partial pressure carbon dioxide (tcPCO_2_) and oxygen saturation (SpO_2_) before and after the subject’s shift to identify changes.

Results: Thirty-nine subjects enrolled in the study (N95 mask = 13, loop/surgical mask = 26). Overall, 69.2% of the subjects continuously wore a mask for five or more hours on their shift. There was a statistical difference with reported fatigue with exclusively wearing an N95 mask versus a loop/surgical mask (p=0.017). None of the other parameters showed a statistical difference between groups. The tcPCO_2_ was not statistically different between mask types at the beginning of the shift (p=0.922) or at the end of the shift (p=0.188), although tcPCO_2_ levels increased. The SpO_2_ was not statistically different between the mask types at the beginning of the shift (p=0.883) or at the end of the shift (p=0.505) with SpO_2_ decreasing.

Conclusion: Individuals exclusively wearing an N95 mask reported a statistically greater number of complaints of fatigue after their shift. No statistical differences were observed in arterial blood gas parameters measured for SpO_2 _and tcPCO_2_ between mask groups. No definitive conclusions can be made due to the small sample size.

## Introduction

The unprecedented novel COVID-19 (SARS-CoV2) pandemic continues to have a catastrophic impact on the global population. The World Health Organization (WHO) declared an infectious disease pandemic on January 30, 2020. To date, according to WHO, there have been over 775.8 million cases and over 7.05 million deaths worldwide, and in the United States, there have been over 103.4 million cases and over 1.2 million deaths as of August 12, 2024 [1]. On May 5, 2023, WHO declared an end to the global public health emergency for COVID-19 [2], and the United States declared the same on May 11, 2023 [3]. However, COVID-19 remains a health threat due to its highly mutative nature.

According to WHO, “Airborne transmission of infectious agents refers to the transmission of disease caused by the dissemination of droplet nuclei that remain infectious when suspended in air over long distance and time.” Several types of respiratory pathogens can be spread via aerosols, such as human coronaviruses, influenza, rhinoviruses, and measles [4]. Two meta-analysis studies investigated the effectiveness of wearing masks to prevent the spread of respiratory viral infections and concluded wearing a mask greatly lowered the risk of transmission [5,6]. Because COVID-19 is spread by respiratory droplets, hospitals developed several guidelines to contain the spread of infection and recommended that healthcare workers routinely use filtering facepiece respirators (FFRs) throughout their entire hospital shift.

Both N95 (non-oil, 95% efficiency) masks and surgical masks are commonly used FFRs. According to the United States Food and Drug Administration (US FDA), N95 masks are personal protective equipment (PPE) to protect wearers from particles or liquids that can contaminate the face. In accordance with FDA standards, an N95 mask indicates that the respirator blocks at least 95% of 300 mm particles. These masks are designed to achieve a very close facial fit to form a seal around the nose and mouth for efficient filtration of airborne particles. Loop/surgical masks are held on the user’s face by loops that go over the ears and are disposable. A loop/surgical mask is made for a loose fit to the facial contour and protects the user only against large droplets [7].

Due to the extremely contagious nature of COVID-19 and other respiratory illnesses, healthcare workers often wear N95 masks and/or surgical masks for much longer durations. In order to maintain conservative numbers of masks used, N95 masks are often worn continuously for prolonged durations versus disposable loop/surgical masks. In particular, operating room staff are required to wear an N95 mask during long surgical cases and airway management due to the higher risk of exposure to aerosolized viruses during procedures. At the end of their long shifts, operating room staff have reported headaches, dizziness, fatigue, exhaustion, and physical and mental discomfort [8].

Wearing N95 masks for longer durations may create significant changes in the microenvironment beneath the mask, such as trapping of heat, moisture, and exhaled carbon dioxide (CO_2_) [9]. Additionally, the use of facemasks potentially hinders normal breathing processes, causing a general thermal discomfort that affects the entire body [10]. Inspiration of trapped exhaled CO_2_ for longer durations can potentially change the dynamics of arterial and venous blood gas parameters, which can result in increased partial pressure carbon dioxide (PCO_2_) (hypercapnia), reduction of partial pressure oxygen (PO_2_), and blood oxygen saturation (SaO_2_), or even more seriously, it can lead to hypoxemia in patients with chronic respiratory problems [9,11]. The change in dynamics of arterial and venous blood gases may be the cause of post-N95 mask blues, and more specifically, the symptoms of fatigue and exhaustion that operating room staff have reported experiencing.

Some studies have analyzed the effects of N95 masks during prolonged durations. Beder et al. [9] analyzed whether a surgeon’s SaO_2_ was affected by a surgical mask during major operations. The study observed that as the duration of the operation increased, SaO_2_ of hemoglobin decreased significantly, especially in surgeons who performed surgeries that were three to four hours in duration [9]. Johnson [12] found that tight-fitting masks can cause inadequate ventilation and increased levels of CO_2_ and noted symptoms of hypoxemia with prolonged mask-wearing duration. They also found the exhaled CO_2_ buildup between the mask and face led to confusion, impaired cognition, and disorientation [12]. In another study, Tong et al. [13] studied the respiratory effects of prolonged respirator use on pregnant women. They observed that breathing through N95 mask materials impeded gaseous exchange and imposed an additional workload on the metabolic system of pregnant healthcare workers [13]. Nafisah et al. [14] observed a significant difference in PCO_2_ levels and PO_2_ levels before and after four hours of continuous use. They also noted significant differences in the levels of venous CO_2_ and oxygen (O_2_). They concluded that there is a risk of hypoxia and a slight increase in CO_2_ concentrations with N95 masks [14]. Kumar et al. found a significant change in SaO_2_ measured with pulse oximeter levels and increased heart rates between the three-hour and six-hour duration of mask usage for N95 masks of 91 pediatric COVID-19 ward healthcare workers [15].

There are no established guidelines for continuous wear of either N95 masks or loop/surgical masks. In a recent study, Guan et al. [16] described an optimal wear time for N95 masks and loop/surgical masks. For filtration of particles and liquid barriers, both mask types appeared to be safe at six hours of use; however, bacteria colonies increased substantially after two hours. In terms of comfort, the inspiratory resistance of both types of masks increased significantly, and the temperature of the face, mouth, and nose that were covered by the masks rose substantially after two hours. They recommended that masks be replaced every two hours if possible [16]. Yang et al. also found that wearing a surgical mask for more than two hours can significantly decrease SPO_2_, and as durations increased, shortness of breath, dizziness, and headaches also increased [17].

Published studies in the literature have analyzed N95 masks’ effects for shorter durations of continuous wear. There is a lack of data on the effects of N95 masks compared to loop/surgical masks on blood gases in healthcare workers who continuously wear these masks for longer durations (up to eight hours) or adverse symptoms.

Because new worldwide viruses and infections, including the continuing mutations of COVID-19, will continue, the use of masks to prevent the spread of respiratory illnesses will be required. It is a certainty that mask usage for prolonged durations will be required in the future within hospitals, workforce, and school settings. The primary purpose of this pilot, observational study was to analyze and compare the effects of wearing N95 masks compared to loop/surgical masks for continuous longer durations on arterial blood gas parameters before and after shifts and any adverse reported symptoms.

This article was previously presented as a paper presentation at the American Academy of Orthopaedic Surgeons (AAOS) 2022 Annual Meeting on March 22-26, 2022.

## Materials and methods

Study design and population sample

This was a non-blinded, pilot, observational study at a single-center, academic institution. Thirty-nine volunteer healthcare workers from the operating room staff at our institution agreed to participate in this study.

Ethical considerations

The Institutional Review Board of the State University of New York Upstate Medical University determined this pilot study was exempt because no subject-identifying information or direct identifier was used or retained. Additionally, the subjects who volunteered provided their verbal and written consent when they agreed to be part of this pilot study.

Study parameters

**Figure 1:**
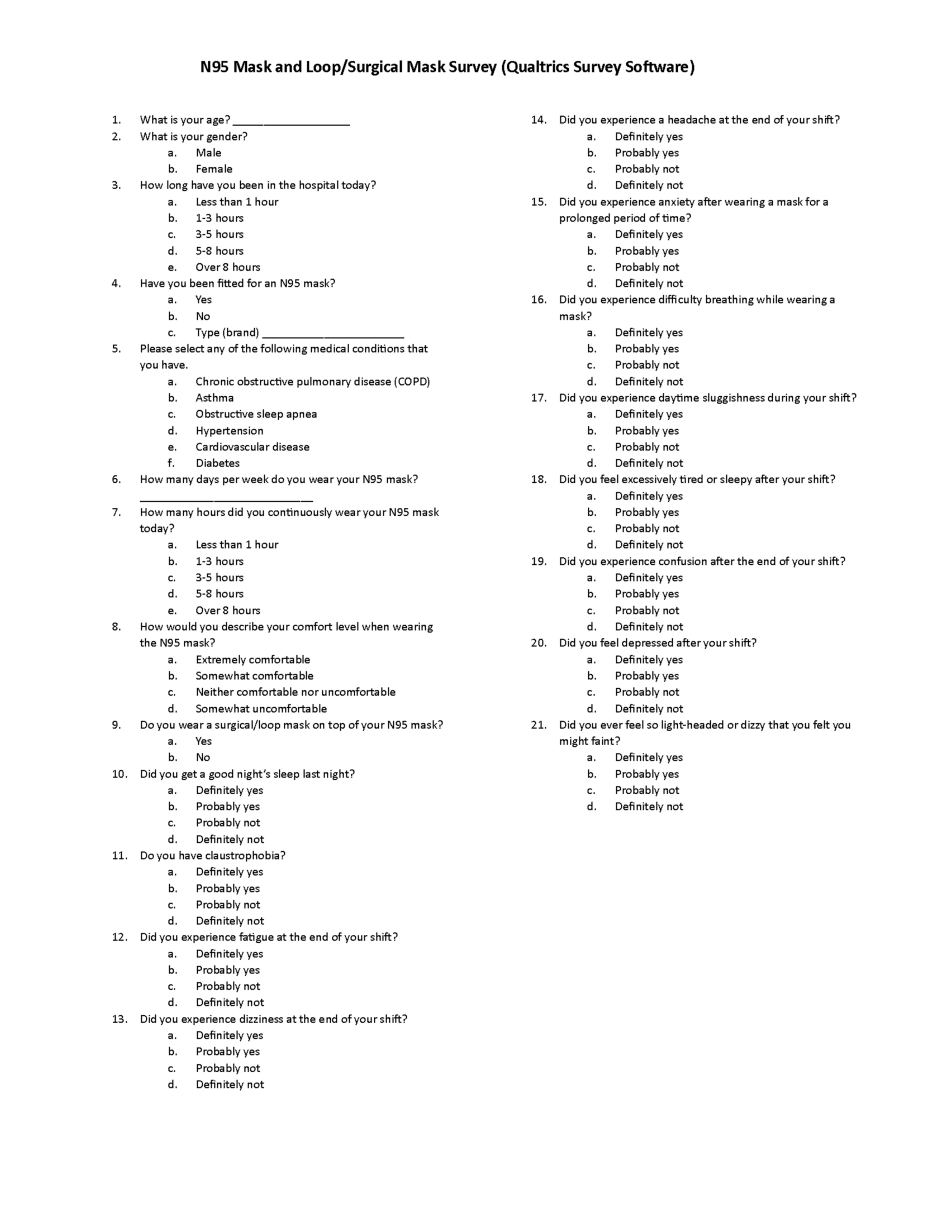
N95 mask and loop/surgical mask survey (Qualtrics survey software) Credit: Authors

We designed a survey from Qualtrics.com (Qualtrics, Seattle, WA, USA) to collect information on age, gender, N95 or loop/surgical mask use, symptomology the subjects experienced, and duration of use. Specific questions related to the subject’s symptomology using a Likert scale were included in the survey. For example, questions related to the frequency and severity of a subject’s fatigue, dizziness, headache, and physical discomfort from the factors mentioned above were included (Figure 1).

Detailed questions on whether the subject was fitted for an N95 mask and the type and manufacturer of the N95 mask were ascertained. Medical co-morbidities specifically related to the subject’s underlying respiratory and cardiac function were included. Information on the subject’s uninterrupted use of the N95 mask was recorded. Because operating room staff are frequently required to wear a surgical mask on top of the N95 mask in order to keep the underlying N95 mask as clean as possible for reuse throughout the day, we questioned whether the subject wore a surgical mask on top of the N95 mask.

Additionally, we measured the transcutaneous partial pressure carbon dioxide (tcPCO_2_) from a tcPCO_2_ monitor and SpO_2_ obtained from a pulse oximeter before and after each subject’s shift to assess any changes in arterial PCO_2_ or changes in SpO_2_ potentially due to re-breathing from the N95 mask or loop/surgical mask. Transcutaneous PCO_2_ is a reliable measurement and estimate of arterial PCO_2_ [18]. The core measurement of tcPCO_2_ involves the production of CO_2_ by tissues, the removal of CO_2 _from tissues by perfusion, and the reference value of CO_2_ at tissue inlet represented by arterial CO_2_ content [18]. This method of measurement of CO_2_ eliminates the need for invasive testing on subjects within the study. A pulse oximeter is a noninvasive way to measure SpO_2_ carried in your red blood cells. It is attached to the subject’s finger and provides a rapid measurement.

Statistical analysis

Descriptive statistics were reported on the subjects from the study, including mean age, morbidity, and gender. ANOVA was utilized for continuous variables. A p-value <0.05 was considered significant.

## Results

Thirty-nine subjects volunteered to take part in this study, with the majority (26/39, 66.67%) preferring to wear only loop/surgical masks compared to only 33.33% (13/39) exclusively wearing N95 mask protection during their shifts. Of the 13 N95 mask wearers, four wore an additional loop/surgical mask over their N95 mask for additional protection. Overall, 38 subjects had been fitted for an N95 mask. The most common type of N95 mask was the Fluidshield duck-billed with 53.84% (N95 = 7, loop/surgical = 14), followed by the 3M 8210 Plus N95/Aura with 25.64% (N95 = 4, loop/surgical = 6), the 3M 18605 NIOSH N95 with 15.38% (N95 = 1, loop/surgical = 5), and one N95 mask wearer with another brand. Twenty-five subjects (64.10%) identified as female. The overall mean age was 39.9 years (N95 = 35.3 years (range 24-58), loop/surgical = 42.2 years (range 24-58)). In total, 12 subjects reported a morbidity (N95 = 3 (2 = asthma and 1 = hypertension); loop/surgical mask = 9 (3 = asthma, 4 = hypertension, 2 = obstructive sleep apnea)). A majority of the subjects in both groups (N = 27, 69.2%) reported continuously wearing their masks for five to eight hours or greater than eight hours. Lastly, when queried about the comfort level of the mask usage, a total of 18 stated somewhat uncomfortable (N95 = 4, loop/surgical = 14), 13 answered neither comfortable nor uncomfortable (N95 = 6, loop/surgical = 7), six answered somewhat comfortable (N95 = 3, loop/surgical = 3), and two wearers of loop/surgical masks stated extremely comfortable (Table 1).

**Table 1:** Comparison of demographics between N95 mask and loop/surgical mask wearers Descriptive statistics were used and ANOVA testing was used to obtain p-values. A p-value <0.05 was considered significant. There were no significant differences observed between N95 mask and loop/surgical mask wearers in the comparison of demographics. ANOVA: analysis of variance

	N95 mask, N=13	Loop/surgical mask, N=26	p-value
Gender			0.033
Female	5 (38.5%)	20 (76.9%)	
Male	8 (61.5%)	6 (23.1%)	
Mean age (years)	35.3 (range: 24-58)	42.2 (range: 22-58)	0.134
Overall morbidity	3 (23.1%)	9 (34.6%)	
Asthma	2 (15.4%)	3 (11.5%)	
Hypertension	1 (7.7%)	4 (15.4%)	
Obstructive sleep apnea	0 (0.0%)	2 (7.7%)	
Hours of continuous wear			
Less than 5 hours	3 (23.1%)	9 (34.6%)	
5 to 8 hours	10 (76.9%)	10 (38.5%)	
Greater than 8 hours	0 (0.0%)	7 (26.9%)	
Comfort level of mask usage			
Somewhat uncomfortable	4 (30.8%)	14 (53.8%)	
Neither comfortable nor uncomfortable	6 (46.1%	7 (26.9%)	
Somewhat comfortable	3 (23.1%)	3 (11.5%)	
Extremely comfortable	0 (0.0%)	2 (7.7%)	

In the category of having a good night’s sleep prior to their shift, there was no statistical difference between the mask groups (p=0.903). Overall, a majority (N = 27, 69.2%) reported either definitely or probably having a good night’s sleep prior to their shift (N95 = 3 and loop/surgical = 4, 17.95% definitely) and N95 = 6, loop/surgical = 14, 51.28% probably). For reported fatigue after a shift, there was a statistical significance (p=0.017) between the mask groups, with 92.31% of subjects using an N95 mask categorically reporting feeling fatigue compared to 65.38% of loop/surgical mask wearers. There were no statistical differences in reported dizziness (p=0.485), headache (p=0.309), or anxiety (p=0.523) after the subjects’ shift between the mask groups. Additionally, other parameters surveyed were being extremely tired at the end of their shift (overall = 23, N95 = 9 and loop/surgical = 14); having difficulty breathing during shift (overall = 23, N95 = 10 and loop/surgical = 13); feeling confused at the end of their shift (overall = 3, N95 = 1 and loop/surgical = 2); or feeling depressed after their shift (overall = 4, N95 = 0 and loop/surgical = 4) (Table 2).

**Table 2:** Comparison of symptoms between N95 mask and loop/surgical mask wearers Descriptive statistics were used, and ANOVA was used to calculate p-values. A p-value <0.05 was considered significant. The only significant factor of symptoms reported between N95 masks and loop/surgical mask wearers was fatigue after shift (p=0.017). ANOVA: analysis of variance

Symptoms	N 95 mask, N=13	Loop/surgical mask, N=26	p-value
Good night’s sleep trend prior to shift			0.903
Probably yes	6 (46.1%)	14 (53.8%)	
Definitely yes	3 (23.1%)	4 (15.4%)	
Probably no	3 (23.1%)	5 (19.2%)	
Definitely no	1 (7.7%)	3 (11.5%)	
Fatigue after shift			0.017
Probably yes	11 (84.6%)	8 (30.8%)	
Definitely yes	1 (7.7%)	9 (34.6%)	
Probably no	0 (0.0%)	3 (11.5%)	
Definitely no	1 (7.7%)	6 (23.1%)	
Dizziness after shift			0.485
Probably yes	1 (7.7%)	3 (11.5%)	
Definitely yes	0 (0.0%)	0 (0.0%)	
Probably no	6 (46.1%)	7 (26.9%)	
Definitely no	6 (46.1%)	16 (61.5%)	
Headache after shift			0.309
Probably yes	4 (30.8%)	9 (34.6%)	
Definitely yes	3 (23.1%)	1 (3.8%)	
Probably no	3 (23.1%)	7 (26.9%)	
Definitely no	3 (23.1%)	9 (34.6%)	
Anxiety after shift			0.523
Probably yes	1 (7.7%)	3 (11.5%)	
Definitely yes	0 (0.0%)	3 (11.5%)	
Probably no	3 (23.1%)	7 (26.9%)	
Definitely no	9 (69.2%)	13 (50.0%)	

The mean tcPCO_2_ measurements at the beginning of the shift were not statistically different between mask types (p=0.922; N95 = 36.54 mmHg (millimeters of mercury) compared to loop/surgical = 36.67 mmHg). The mean tcPCO_2_ measurement at the end of the shift was also not statistically different between mask types (p=0.188; N95 = 37.35 mmHg compared to loop/surgical = 37.23 mmHg). Of note, from the beginning to the end of the shift, tcPCO_2_ levels increased by 0.81 mmHg for N95 mask wearers and 0.56 mmHg for loop/surgical mask wearers. The mean SpO_2_ reading at the beginning of the shift was not statistically different between the mask types (p=0.883; N95 = 98.41% compared to loop/surgical = 98.36%). Also, the mean SpO_2_ reading at the end of the shift was not statistically different between mask types (p=0.505; N95 = 97.77% compared to loop/surgical = 98.08%). However, from the beginning to the end of the shift, SpO_2_ levels decreased by 0.64% for N95 masks and 0.26% for loop/surgical masks (Table 3).

**Table 3:** Comparison of tcPCO2 and SpO2 with pulse oximeter between N95 mask and loop/surgical mask usage Descriptive statistics were used to calculate mean values. ANOVA was utilized to obtain p-values. A p-value <0.05 was considered significant. There were no significant differences measured for tcPCO2 and SPO2 between N95 mask and loop/surgical mask wearers. tcPCO_2_: transcutaneous partial pressure carbon dioxide, SpO_2_: oxygen saturation, ANOVA: analysis of variance

	N95 mask, N=13	Loop/surgical mask, N=26	p-value
Mean tcPCO_2 _beginning of shift (mmHg (millimeters of mercury))	36.54	36.67	0.922
Mean tcPCO_2_ end of shift (mmHg)	37.35	37.23	0.188
Mean SpO_2_ beginning of shift (%)	98.41	98.36	0.883
Mean SpO_2_ end of shift (%)	97.77	98.08	0.5052

## Discussion

This was a pilot, observational study that compared continuously wearing N95 masks versus loop/surgical masks for long durations and observed no significant effects on arterial gases in our subjects through tcPCO_2_ measurements and SpO_2_ readings. In both groups, from the beginning to the end of the shift, tcPCO_2_ levels increased and SpO_2_ levels decreased. We also queried the subject’s symptomology using a Likert scale to assess comparisons between the mask groups for fatigue, dizziness, headache, anxiety, depression, confusion, physical discomfort, and sleeping trends. The only symptom that was observed to be significant was reported fatigue with N95 mask wearers compared to loop/surgical mask wearers.

PCO_2_ measurements for CO_2_ levels

The measurement of PCO_2_ in arterial blood remains the gold standard for the assessment of ventilation. The tcPCO_2_ monitor is considered a valid noninvasive method in routine respiratory care to assess the adequacy of ventilation [18]. The normal range for tcPCO_2_ measurements is 35 to 45 mmHg [19]. In our study, no statistical differences between mask types were noted at the beginning (p=0.922) or the end (p=0.188) of the subject’s shift. The high disparity in p-values is probably not significant due to our small sample size. It is important to note the tcPCO_2_ levels remained within the normal range at both time points. Hypercapnia occurs when the CO_2 _blood level rises above the normal range due to respiration. We found CO_2_ levels increased by 0.81 mmHg for N95 masks and 0.56 mmHg for loop masks from the beginning to the end of the shift; however, these levels also remained within the normal range. Although our study did not demonstrate significance, we observed similar findings of increased CO_2_ levels that were reported by Johnson et al. [12] and Nafisah et al. [14].

SpO_2_ levels

Pulse oximetry is a convenient, noninvasive method utilized to determine SpO_2_. Pulse oximeters have a high accuracy in the detection of hypoxia (values <90%). Normal SpO_2_ readings should range between 95% and 100%. We observed that there were no significant differences between N95 and loop/surgical mask wearers at either the beginning (p=0.883) or end (p=0.505) of their shifts. Our study found that mean SpO_2_ levels decreased for both groups during their mask-wearing duration but remained within the normal range. In concurrence with Beder et al. [9], surgical mask wearers showed SpO_2_ levels decreased. Although we observed only slight changes in SpO_2_ wearing an N95 mask, Kumar et al. [15] observed significant changes.

Rebmann et al. [20] observed that CO_2_ levels increased, and there were no dynamic changes in SpO_2_ compared to baseline measures [20]. In another study of 75 healthcare professionals, the authors were unable to find substantial data highlighting the retention of noxious gases and their effect on respiratory function with the use of PPE [21]. Nwosu et al. found no significant change in arterial SpO_2_ between N95 and surgical mask wearers [22]. Spang and Pieper [23] measured the SpO_2_ of 44 participants both wearing and not wearing N95 masks. They found no significant differences and only a slight, non-significant decrease of 0.3% in SpO_2_ levels after wearing an N95 mask [23]. Similarly, Singla et al. found no significant drop in SpO_2_ from the start of a shift to six hours of duration in 109 intensive care staff and healthcare workers for N95 mask usage [24].

Su et al. also found no significant changes in physiological parameters (SpO_2_, PO_2_, PCO_2_, blood pressure, and peripheral pulse rate) and concluded the N95 group did not show a higher risk of any harmful effects on the health of their workers [7]. Lim et al. found that CO_2_ levels may increase after the one-hour mark, which could affect the health of the wearer [25]. Our study produced similar results that were observed in these studies, with a non-significant increase in CO_2_ levels or a decrease in SpO_2_ levels for both mask groups.

Symptomology parameters

From our questionnaire about symptomology, the only significant difference between wearing N95 masks and loop/surgical masks was reported fatigue (p=0.017) with 92.31% of N95 mask wearers. We observed no significant differences with any other queried symptom between N95 or loop/surgical mask usage. Rebmann et al. concluded that long-term use of respiratory protection did not result in any clinically relevant physiologic burden, although many subjective symptoms were reported [20].

Conversely, many studies have observed significant symptoms. Athar et al.'s study of 75 subjects found 70 experienced headaches, 50 lethargy, 38 dizziness, 23 nausea, 18 dyspnea, and 12 tachypnea [21]. Su et al.'s study of 68 healthcare workers compared N95 respirators and surgical masks for an eight-hour shift in the emergency department. The N95 group had significantly higher reports of shortness of breath, headaches, dizziness, difficulty talking, and fatigue [7]. Lim et al. observed that 37.3% of healthcare workers who wore an N95 mask for more than four hours reported having headaches [25]. Another study of 343 healthcare professionals wearing either an N95 mask (59.2%) or a surgical mask found that 71.4% reported headaches from prolonged mask use [26]. Shubhanshu and Singh surveyed 423 healthcare workers (67% = N95 mask and 33% = surgical mask) and found that headaches were the most common symptom of wearing a mask for prolonged durations and found no significant changes in other parameters [27].

Psychologically, chronic stress after long-term wearing of N95 or surgical masks may affect the body and cause headaches, dizziness, mood disturbances, and a reduction in cognitive performance [28,29]. Su et al. [7] also hypothesized that the higher incidence of shortness of breath, headaches, dizziness, difficulty talking, and fatigue in the N95 group versus the surgical mask group may originate from psychological factors. They found the symptoms gradually disappeared over time [7]. Rebmann et al. observed that healthcare workers adapted to wearing N95 masks increased over time [20]. A confounding factor in our study was that the only symptom of significance was fatigue after a shift. Perhaps the operating room staff had already adapted to wearing masks for long durations over time. Also, our subjects’ reported fatigue may be due to the stressful times during the initial stages of COVID-19. Therefore, the benefits of wearing an N95 mask or loop/surgical mask to reduce the spread of viral respiratory diseases should be weighed against any consequences associated with extended wearing durations.

Limitations

There were several limitations in our study. It was a pilot, observational, non-blinded, voluntary study conducted at a single academic institution, resulting in a small sample size. All participants were volunteers, which may have limited the diversity of the sample and affected the reproducibility of the results. Another limitation could be the accuracy of tcPCO_2_ and SpO_2_ measurements obtained using pulse oximeters. Since this was a pilot study, no meaningful data were available to link chronic daily fatigue with tcPCO_2_ levels. Additionally, the survey instrument used was not a previously validated tool. However, a strength of our study was that longer continuous durations of mask usage produced similar findings to previous studies that investigated shorter durations. This could be important for healthcare workers who wear masks for extended periods, as it suggests they might not be adversely affected.

The next step in our research is to conduct a study with a larger sample of various types of healthcare workers within our institution, incorporating additional parameters (BMI, heart rate, blood pressure, etc.). Further studies are necessary to gather data from more institutions and examine the long-term effects of prolonged mask use. Additional research could also include the general population outside of a hospital setting. Moreover, a comprehensive review of existing studies on the effects of mask wearing could be initiated to consolidate the findings.

## Conclusions

Individuals wearing an N95 mask reported a significantly greater number of complaints of fatigue after their shift despite reporting a good night’s sleep prior to their shifts. There were no statistical differences observed in arterial blood gas parameters between transcutaneous measured values of SpO_2_ and tcPCO_2_ between mask groups for long durations of continuous mask usage. No definitive conclusions can be made due to the small sample size; therefore, further studies are needed to clarify the results. Due to the cyclical nature of viruses, it is a certainty that mask usage for prolonged durations may be required in the future for the entire global population.
